# RECRUITMENT FOR A MULTISITE PRAGMATIC TRIAL OF DEMENTIA CARE STRATEGIES: BARRIERS AND SUCCESS DURING COVID-19

**DOI:** 10.1093/geroni/igac059.1207

**Published:** 2022-12-20

**Authors:** David Reuben, Katie Maslow

## Abstract

With 2176 participants recruited, D-CARE is the largest pragmatic clinical trial of dementia care strategies, to date. At four clinical trial sites (CTS), D-CARE will compare the effectiveness of three dementia care strategies over 18 months: 1) by nurse practitioners or physician assistants within a health care system, 2) by social workers or nurses at community-based service organizations (CBO), or 3) usual care. Primary outcomes include person with dementia (PWD) behavioral symptoms and caregiver strain. Other outcomes include the PWD quality of life and ability to reach personal goals, and caregiver self-efficacy, distress, and depressive symptoms. Recruitment began in June 2019 with a basic protocol in which participating providers reviewed lists generated from the electronic health records (EHR) of patients who had a diagnosis of dementia, allowing the removal of patients who should not be contacted and giving an opportunity to provide information about the family caregiver. Some practices gave “blanket” referral allowing research staff to recruit participants directly. Other practices provided direct referrals via EHR communications to the research team. Self-referrals triggered by public postings in clinics and CBOs, social media, and media coverage were also accepted if a dementia diagnosis was confirmed in the EHR. By March 16, 2020, all in-person recruitment visits were suspended due to COVID-19. In response, informed consent was switched to telephone with verbal consent as permitted by State and Institutional regulations. This symposium describes the creative approaches employed by CTS’ to respond to these challenges and reach the recruitment goal in January 2022.

